# Association between food insecurity and kidney stones in the United States: Analysis of the National Health and Nutrition Examination Survey 2007–2014

**DOI:** 10.3389/fpubh.2022.1015425

**Published:** 2022-11-09

**Authors:** Wei Wang, Xi Lu, Yixiao Shi, Xin Wei

**Affiliations:** ^1^Department of Urology, Institute of Urology, West China Hospital, Sichuan University, Chengdu, China; ^2^Department of Radiology, West China Hospital, Sichuan University, Chengdu, China; ^3^Operating Room of Anesthesia Surgery Center, West China Hospital/West China School of Nursing, Sichuan University, Chengdu, China

**Keywords:** food insecurity, kidney stones, nephrolithiasis, NHANES, cross-sectional study

## Abstract

**Purpose:**

Although food insecurity is a major public health concern associated with various diseases, the relationship between food insecurity and kidney stones remains unclear. We aimed to investigate the association between food insecurity and kidney stones in the US population.

**Materials and methods:**

Four continuous cycles of data from the year 2007 to 2014 were obtained from National Health and Nutrition Examination Survey (NHANES) in the current study. We utilized the Household Food Security Module to assess the food security status of individuals. The primary outcome was whether participants ever had kidney stones, and the secondary outcome was a stone recurrence. A multivariate logistic regression model adjusting for potential confounders was constructed to evaluate the independent association between food insecurity and kidney stones.

**Results:**

A total of 21,914 participants were included in our analysis, with 8.8% having a history of kidney stones and 33.2% of these reporting stones recurrence. Food insecurity was associated with increased risks of kidney stones (odds ratio: 1.21; 95% confidence interval: 1.05–1.39; *P* = 0.010) and kidney stones recurrence (odds ratio: 1.33; 95% confidence interval: 1.00–1.77; *P* = 0.052) after adjusting for all potential confounders. In addition, participants with very low food security had 38% and 47% higher risks of kidney stones (odds ratio: 1.38; 95% confidence interval: 1.13–1.69; *P* = 0.002; *P* for trend = 0.009) and kidney stones recurrence (odds ratio: 1.47; 95% confidence interval: 1.03–2.10; *P* = 0.032; *P* for trend = 0.029), respectively.

**Conclusion:**

There exists a significant association between food insecurity and kidney stones, which reveals the significance of the improvement of food insecurity in the alleviation of kidney stone formation and recurrence.

## Introduction

Kidney stones are a common disease in the urinary system and are caused by the abnormal crystallization of minerals. The prevalence rate of kidney stones has increased globally over the past few decades. According to the data from the National Health and Nutrition Examination Survey (NHANES), the self-reported prevalence of kidney stones has increased from 5.2% in the period 1988–1994 to 8.8% in 2007–2010 (1, 2). The lifetime prevalence of kidney stones was estimated to be 10.1% in the United States (U.S.), 5–10% in Europe, 4% in South America, and 1–19% in Asia (3, 4). Moreover, kidney stones also demonstrate an ~50% of recurrence rate at 10 years (3). Thus, kidney stones represent a huge public health burden and the annual expenditure exceeded $2 billion in the U.S. alone (5). The propensity of stones formation differs according to sex and the male-to-female ratio of kidney stones was estimated to be 1.3 (6). In addition, environmental (e.g., hot and arid climates) and diseases (e.g., obesity and diabetes) factors are positively associated with kidney stone formation (6). For example, Scales et al. demonstrated that obesity and diabetes were associated with 55 and 59% higher risks of developing kidney stones after adjusting for potential covariates (2).

Food insecurity refers to the lack of physical, social, and economic access to adequate nutritious food (7). Food insecurity is a major public health concern that occurs not only in developing countries but also in developed countries such as the U.S. (8). It was reported that 10.5% of U.S. households experienced food insecurity in 2019. This rate has dramatically increased to 38.3% since the outbreak of the novel coronavirus disease 2019 (COVID-19) (9). A recent study suggested that 44% of U.S. adults below 250% of the federal poverty line were food insecure in 2020 (10). Furthermore, the COVID-19 pandemic has exacerbated the existing disparities among already at-risk individuals (10). Those who have food insecurity are more likely to consume cheaper, high-energy-dense, sodium-rich diets as a result of limited resources. Increasing evidence shows food insecurity is related to many health-related problems such as obesity, diabetes, cardiovascular disease, hypertension, erectile dysfunction, depression, and kidney disease (8–12). Food insecurity might also be positively associated with kidney stones via its effects on the risk factors for stones (e.g., obesity and diabetes) or the direct impacts of diet on the stones. For instance, men with food insecurity consuming a sodium-rich diet might increase urinary calcium, reduce urinary citrate and raise urate saturation, all of which lead to calcium oxalate precipitation, and finally result in increased risks of developing kidney stones (13). A retrospective case-control study with a small sample size determined the relationships between food insecurity and stone formation (14). The cases were diagnosed with calcium oxalate stones by a urologist but without other diseases including diabetes, hypertension and hyperlipidemia. The controls were matched by gender, age, and place of residence. Their results showed that 68% of cases and 40% of controls were food insecure, respectively (14). However, the study only included 200 individuals for analysis and they fail to determine the associations between food insecurity and stone recurrence. Therefore, we obtained data from the 2007–2014 cycles of the NHANES, for the first time, to determine whether food insecurity can increase the risk of kidney stones. Moreover, the association between food insecurity and stone recurrence was also assessed in the current study.

## Methods

### Study population

**Figure 1:**
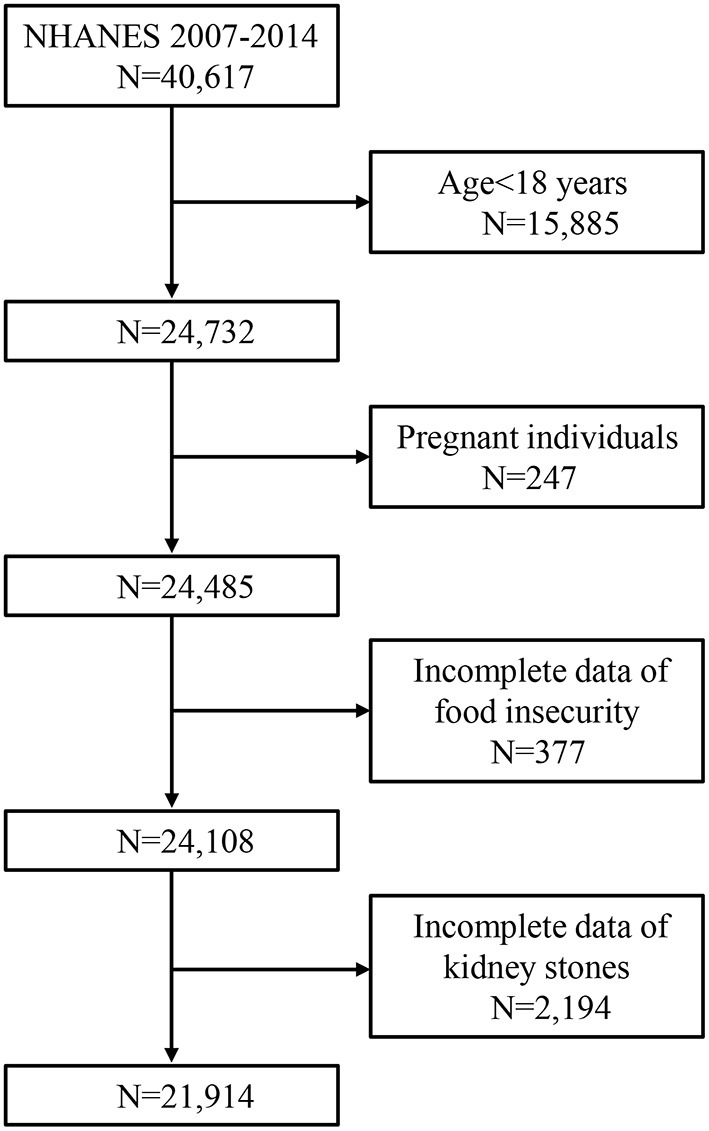
Flowchart of the study population from NHANES 2007–2014.

NHANES is one of a series of nationwide surveys and physical examinations undertaken by the Centers for Disease Control and Prevention's National Center for Health Statistics (NCHS) in the United States. The NHANES study surveys collected various health and nutritional data from samples of the resident civilian adults and children utilizing a kind of complex, multistage probability sampling design. Four continuous cycles of data from the year 2007 to 2014 were obtained from NHANES in the current study, as the data regarding kidney stones and kidney stones recurrence is only recorded in these years. The individuals with incomplete information regarding kidney stones and food insecurity were excluded from our study. Ultimately, 21,914 participants were enrolled in the final analysis. The detailed flowchart of sample selection was presented in Figure 1. The study protocols were granted by the ethics review board of the NCHS, and informed consent was signed by each participant.

### Exposure and outcome definitions

We utilized the Household Food Security Module to assess the food security status of participants. This module includes 10 or 18 questions specific to the food security-related condition of households without or with children, respectively. The questions were detailedly shown in Supplementary Table 1. A score ranging from 0 to 10 was obtained by a summation of the affirmative responses of participants. The lower score indicated less food insecurity. Food insecurity was categorized as low (scores 3–5) or very low food security (score 6–10), while full (score 0) or marginal food security (score 1–2) were considered as food security (12, 15–18).

The primary outcome was whether participants ever had kidney stones, which was evaluated by the question, “Have you ever had a kidney stone?” Those who responded “yes” were considered to have kidney stones. The secondary outcome was a stone recurrence, as determined by responding to the question “How many times have you passed a kidney stone?” If one person has experienced two or more stones, he/she was regarded as having the recurrence of kidney stones. The validity and accuracy of this self-reported questionnaire have been confirmed and could identify 97% of the men with clinically diagnosed kidney stones (19).

### Covariates

Potential confounders were identified according to previous studies (20, 21): age, gender (male and female), race (Mexican American, other Hispanic, non-Hispanic white, non-Hispanic black, and other race), the ratio of family income to poverty (PIR) (<1.3, 1.3 to 3.5, and >3.5) (22), an education level (less than high school, high school and above high school), marital status (married or living with a partner and living alone), body mass index (BMI), smoking status (non-smoker, former smoker and current smoker), physical activity status (active and inactive), diabetes (Yes and no), serum uric acid, sugar intake, water intake, and Vitamin B6 intake. Individuals were regarded as current smokers if they responded “Yes” to the question “Have you smoked at least 100 cigarettes in your entire life?” and “Yes” to “Do you now smoke cigarettes?” Former smokers were those who responded “Yes” to the first question and “No” to the second question; Participants who answered “No” to the first question were considered non-smokers. Physical activity status was evaluated by whether participants' work involved at least 10 continuous minutes of moderate or vigorous activity (active) or no (inactive) in an average week. Individuals with a previous diagnosis of diabetes were deemed as having diabetes. The Beckman Synchron LX20 was used to measure the concentration of uric acid in serum. Data regarding the total intake of sugars (gm), water (gm), and Vitamin B6 (gm) were collected by the 24-h recall dietary interviews.

### Statistical analysis

Continuous and categorical variables were described by mean ± standard error (SE) and proportions, respectively. The study analysis was adjusted for sampling weights, strata, and primary sampling units. Characteristics for the sample participants between food-secure and food-insecure groups were compared by *t*-test and chi-square test. We constructed two multivariate logistic regression models to evaluate the independent association between food insecurity and kidney stones: (1) In the model I, because of the correlation between demographic variables and kidney stones, we adjusted the age, gender, race, and BMI; (2) In the model II, we further adjusted for the PIR, education level, marital status, BMI, smoking, physical activity, diabetes, serum uric acid, sugar intake, water intake, and Vitamin B6 intake. The Box-Tidwell method was used to evaluate a linear relationship between continuous independent variables and dependent variable logit conversion values (23). Multicollinearity among independent variables was examined by tolerance and variance inflation factor, and there was no interaction. Results were presented as adjusted odds ratios (OR) and 95% confidence intervals (CI).

We also conducted additional sensitivity analyses to assess the robustness of our results. We categorized food security into 4 groups (high, marginal, low and very low) to examine whether there existed a dose-response association between the increasing severity of food insecurity and kidney stones. Missing values were replaced by the median (continuous) or mode (categorical) of existing cases of that variable. All analyses were conducted utilizing the statistical R software (version 4.1.2) and Empower (www.empowerstats.com). Results were statistically significant for *P* < 0.05 (double-sided).

## Results

A total of 21,914 participants were included in our analysis, with 8.8% having a history of kidney stones and 33.2% of these reporting stones recurrence. The baseline characteristics of participants by food security status were shown in Table 1. Compared with the food-secure group, participants in the food-insecure group were more likely to be younger, female, Mexican American, with a PIR <1.3, less educated, living alone, with a higher BMI, current smoker, physically inactive, with a lower level of serum uric acid and a higher sugar intake (all *P* < 0.05) (Table 1).

**Table 1:** Baseline characteristics of participants by food security status in NHANES 2007–2014.

	**Food secure**	**Food insecure**	***P*-value**
Number	17,751	4,163	
Age, years	50.5 ± 0.1	44.1 ± 0.2	<0.001
**Gender**, ***n*** **(%)**			0.005
Male	8,693 (49.0%)	1,938 (46.6%)	
Female	9,058 (51.0%)	2,225 (53.4%)	
**Race**, ***n*** **(%)**			<0.001
Mexican American	2,301 (13.0%)	924 (22.2%)	
Other Hispanic	1,630 (9.2%)	569 (13.7%)	
Non-Hispanic White	8,269 (46.6%)	1,349 (32.4%)	
Non-Hispanic Black	3,626 (20.4%)	1,053 (25.3%)	
Other race	1,925 (10.8%)	268 (6.4%)	
**Ratio of family income to poverty**, ***n*** **(%)**			<0.001
<1.3	4,252 (24.0%)	2,537 (60.9%)	
1.3–3.5	6,129 (34.5%)	1,172 (28.2%)	
Over 3.5	5,897 (33.2%)	151 (3.6%)	
Missing	1,473 (8.3%)	303 (7.3%)	
**Education level**, ***n*** **(%)**			<0.001
Less than high school	3,996 (22.5%)	1,734 (41.7%)	
High school	3,912 (22.0%)	1,085 (26.1%)	
Above high school	9,820 (55.3%)	1,341 (32.2%)	
Missing	23 (0.1%)	3 (0.1%)	
**Marital status**, ***n*** **(%)**			<0.001
Married or living with a partner	10,794 (60.8%)	2,051 (49.3%)	
Living alone	6,948 (39.1%)	2,109 (50.7%)	
Missing	9 (0.1%)	3 (0.1%)	
BMI, kg/m^2^	28.7 ± 0.1	29.9 ± 0.1	<0.001
**Smoking**, ***n*** **(%)**			<0.001
Non-smoker	10,114 (57.0%)	1,957 (47.0%)	
Former smoker	4,443 (25.0%)	726 (17.4%)	
Current smoker	3,194 (18.0%)	1,480 (35.6%)	
**Physical activity status**, ***n*** **(%)**			<0.001
Active	8,596 (48.4%)	1,604 (38.5%)	
Inactive	9,155 (51.6%)	2,559 (61.5%)	
**History of diabetes**, ***n*** **(%)**			0.171
Yes	2,223 (12.5%)	554 (13.3%)	
No	15,528 (87.5%)	3,609 (86.7%)	
Serum uric acid, mg/dl	5.5 ± 0.01	5.4 ± 0.02	<0.001
Sugar intake, gm	111.4 ± 0.6	126.7 ± 1.54	<0.001
Water intake, gm	1029.4 ± 8.4	1033.9 ± 19.3	0.826
Vitamin B6 intake, gm	2.1 ± 0.01	2.0 ± 0.03	0.071

BMI, body mass index.

The values are presented as weighted means ± SE or unweighted counts (weighted %).

The baseline characteristics of participants with or without a history of kidney stones were presented in Table 2. The kidney stones group tended to be older and were more likely to be male, non-Hispanic white, married or living with a partner, having a higher BMI, being a former smoker, lacking exercise, having a history of diabetes, with a higher level of serum uric acid, consuming less water and vitamin B6 (all *P* < 0.05). Additionally, Participants with a history of stone recurrence were more likely to be male, non-Hispanic white, and consuming more sugar (all *P* < 0.001) (Table 2).

**Table 2:** Baseline characteristics of participants by kidney stones in NHANES 2007–2014.

	**History of kidney stones (*****n*** = **21,914)**			**History of stones recurrence (*****n*** = **1,929)**		
	**No**	**Yes**	***P*-value**	**No**	**Yes**	***P*-value**
Number	19,985	1,929		1,289	640	
Age, years	48.7 ± 0.1	56.0 ± 0.4	<0.001	56.1 ± 0.5	55.7 ± 0.6	0.633
**Gender**, ***n*** **(%)**			<0.001			0.016
Male	9,563 (47.9%)	1,068 (55.4%)		689 (53.5%)	379 (59.2%)	
Female	10,422 (52.1%)	861 (44.6%)		600 (46.5%)	261 (40.8%)	
**Race**, ***n*** **(%)**			<0.001			<0.001
Mexican American	2,981 (14.9%)	244 (12.6%)		181 (14.0%)	63 (9.8%)	
Other Hispanic	1,993 (10.0%)	206 (10.7%)		123 (9.5%)	83 (13.0%)	
Non-Hispanic White	8,514 (42.6%)	1,104 (57.2%)		693 (53.8%)	411 (64.2%)	
Non-Hispanic Black	4,430 (22.2%)	249 (12.9%)		198 (15.4%)	51 (8.0%)	
Other race	2,067 (10.3%)	126 (6.5%)		94 (7.3%)	32 (5.0%)	
**Ratio of family income to poverty**, ***n*** **(%)**			0.542			0.586
<1.3	6,191 (31.0%)	598 (31.0%)		402 (31.2%)	196 (30.6%)	
1.3–3.5	6,633 (33.2%)	668 (34.6%)		434 (33.7%)	234 (36.6%)	
Over 3.5	5,536 (27.7%)	512 (26.5%)		352 (27.3%)	160 (25.0%)	
Missing	1,625 (8.1%)	151 (7.8%)		101 (7.8%)	50 (7.8%)	
**Education level**, ***n*** **(%)**			0.214			0.542
Less than high school	5,211 (26.1%)	519 (26.9%)		356 (27.6%)	163 (25.5%)	
High school	4,532 (22.7%)	465 (24.1%)		301 (23.4%)	164 (25.6%)	
Above high school	10,217 (51.1%)	944 (48.9%)		631 (49.0%)	313 (48.9%)	
Missing	25 (0.1%)	1 (0.1%)		1 (0.1%)	0 (0.0%)	
**Marital status**, ***n*** **(%)**			<0.001			0.129
Married or living with a partner	11,613 (58.1%)	1,232 (63.9%)		804 (62.4%)	428 (66.9%)	
Living alone	8,362 (41.8%)	695 (36.0%)		484 (37.5%)	211 (33.0%)	
Missing	10 (0.1%)	2 (0.1%)		1 (0.1%)	1 (0.2%)	
BMI, kg/m^2^	28.8 ± 0.05	30.3 ± 0.15	<0.001	30.2 ± 0.19	30.5 ± 0.27	0.258
**Smoking**, ***n*** **(%)**			<0.001			0.100
Non-smoker	11,114 (55.6%)	957 (49.6%)		660 (51.2%)	297 (46.4%)	
Former smoker	4,577 (22.9%)	592 (30.7%)		389 (30.2%)	203 (31.7%)	
Current smoker	4,294 (21.5%)	380 (19.7%)		240 (18.6%)	140 (21.9%)	
**Physical activity status**, ***n*** **(%)**			<0.001			0.729
Active	9,448 (47.3%)	752 (39.0%)		506 (39.3%)	246 (38.4%)	
Inactive	10,537 (52.7%)	1,177 (61.0%)		783 (60.7%)	394 (61.6%)	
**History of diabetes**, ***n*** **(%)**			<0.001			0.698
Yes	2,347 (11.7%)	430 (22.3%)		284 (22.0%)	146 (22.8%)	
No	17,638 (88.3%)	1,499 (77.7%)		1,005 (78.0%)	494 (77.2%)	
Serum uric acid, mg/dl	5.4 ± 0.01	5.7 ± 0.03	<0.001	5.7 ± 0.04	5.7 ± 0.04	0.789
Sugar intake, gm	114.2 ± 0.6	115.9 ± 1.9	0.380	112.5 ± 2.2	122.7 ± 3.5	0.012
Water intake, gm	1037.5 ± 8.1	956.1 ± 24.5	0.004	936.7 ± 29.9	994.9 ± 42.6	0.276
Vitamin B6 intake, gm	2.1 ± 0.01	2.0 ± 0.03	0.029	2.0 ± 0.04	1.9 ± 0.05	0.423

BMI, body mass index.

The values are presented as weighted means ± SE or unweighted counts (weighted %).

**Figure 2:**
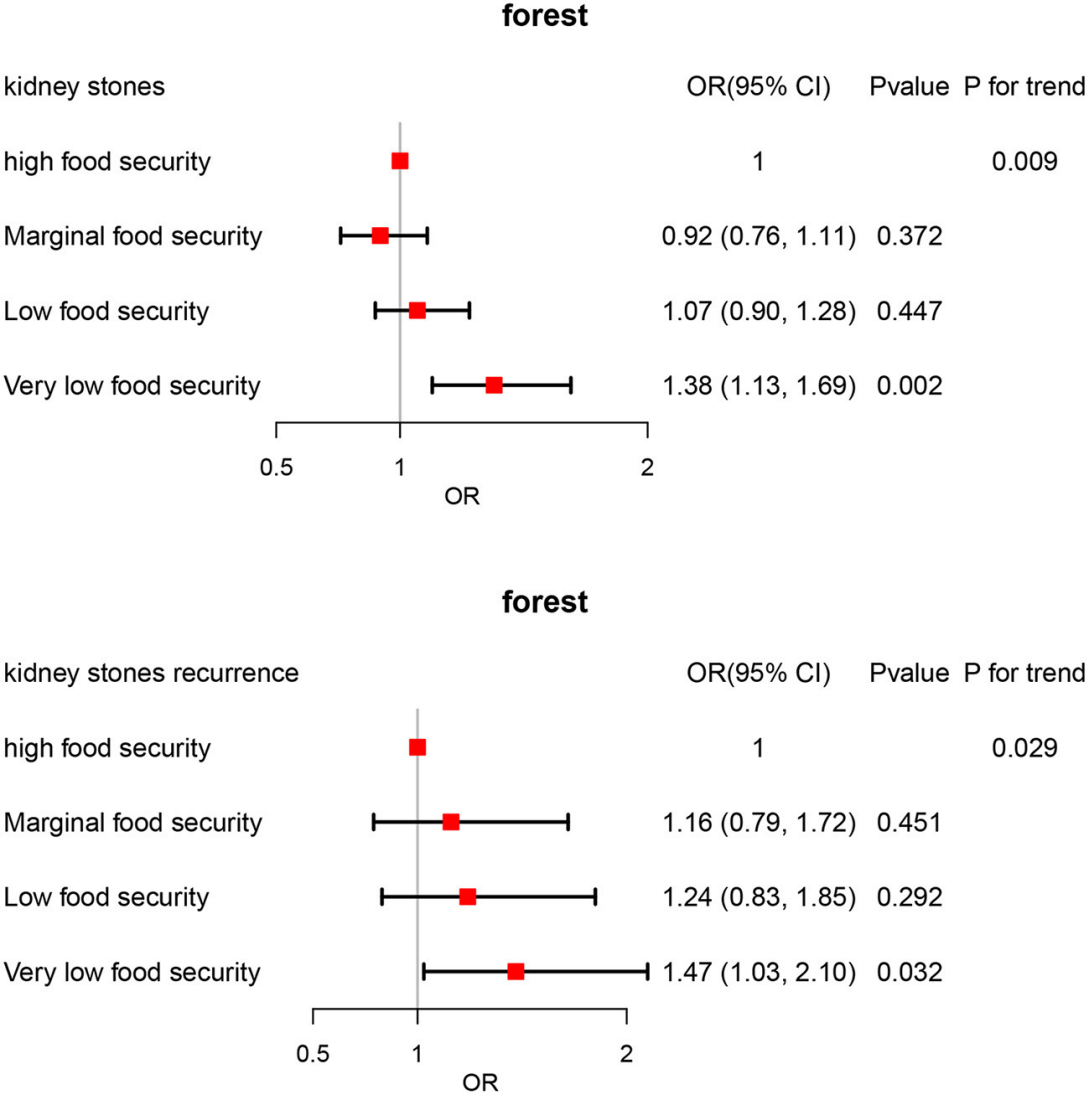
ORs and 95% CIs for kidney stones associated with food insecurity.

As shown in Table 3, food insecurity was significantly associated with kidney stones after adjusting for all potential confounders (Model II, OR 1.21; 95% CI 1.05–1.39; *P* = 0.010); In addition, individuals with food insecurity had a 33% increased risks of stones recurrence after full adjustment although this association did not reach statistical significance (Model II, OR 1.33; 95% CI 1.00–1.77; *P* = 0.052). We further performed sensitivity analysis by categorizing food insecurity into 4 groups. Our result revealed a dose-response association between food insecurity and kidney stones after adjusting for all confounding variables. Participants with very low food security had 38% higher risks of kidney stones compared with those having high food security (Model II, OR 1.38; 95% CI 1.13–1.69; *P* = 0.002; *P* for trend = 0.009) (Figure 2); Moreover, a dose-response association between food insecurity and kidney stones recurrence was also observed after full adjustment. Individuals with very low food security had 47% greater risks of experiencing stones recurrence (Model II, OR 1.47; 95% CI 1.03–2.10; *P* = 0.032; *P* for trend = 0.029) (Figure 2).

**Table 3:** Association between food insecurity and kidney stones in NHANES 2007–2014.

	**OR (95% CI)**, ***P*****-value**	
	**Model I**	**Model II**
Kidney stones	
Low/very low food security (reference: high/marginal)	1.25 (1.11, 1.42), <0.001	1.21 (1.05, 1.39), 0.010
Kidney stones recurrence	
Low/very low food security (reference: high/marginal)	1.41 (1.10, 1.81), 0.007	1.33 (1.00, 1.77), 0.052

Model I: adjust for age, gender, race, and BMI.

Model II: adjust for age, gender, race, PIR, education level, marital status, BMI, smoking, physical activity, diabetes, serum uric acid, sugar intake, water intake, and Vitamin B6 intake.

## Discussion

This is the first large cross-sectional study determining the association between food insecurity and kidney stones. Our results revealed that food insecurity, especially very low food security, was independently related to a higher likelihood of kidney stones and kidney stones recurrence. These positive associations were independent of potential covariates including demographics, socioeconomic factors, physical activity, diabetes, serum uric acid, intake of sugar, water, and vitamin B6. Our study identified food insecurity as a new modifiable risk factor for kidney stones and kidney stones recurrence, highlighting applying public policies to improve the accessibility and quality of food.

Our results showed that food insecurity was associated with kidney stones (OR 1.21; 95% CI 1.05–1.39; *P* = 0.010) and kidney stones recurrence (OR 1.33; 95% CI 1.00–1.77; *P* = 0.052) after adjusting for all potential confounders. Furthermore, we conducted a sensitivity analysis by categorizing food insecurity into 4 groups. Participants with very low food security had 38% higher risks of kidney stones compared with those having high food security (OR 1.38; 95% CI 1.13–1.69; *P* = 0.002; *P* for trend = 0.009). In addition, Individuals with very low food security had 47% greater risks of experiencing stones recurrence (OR 1.47; 95% CI 1.03–2.10; *P* = 0.032; *P* for trend = 0.029). This might be explained by several reasons. Firstly, food-insecure adults consumed fewer fruits, vegetables, and micronutrients when compared to the food-secure group. Diets with a variety of nutritious fruits and vegetables play a critical role in the prevention and management of urolithiasis (24). For example, organic anions associated with potassium in fruits and vegetables can reduce the excretion of urinary calcium (25). Secondly, those who experienced food insecurity consumed more added sugars and sugar-sweetened soft drinks in their daily life (26). These soft drinks contain large amounts of fructose which can lead to an increase in uric acid levels and the frequency of hyperuricemia (27). Additionally, higher intakes of sugar-sweetened soft drinks were also related to an increased risk of gout (28). Thirdly, a growing body of evidence has suggested that food insecurity is a risk factor for depression and stress (29). A systematic review conducted by Pourmotabbed et al. revealed that food insecurity had a significant effect on the likelihood of being stressed in adults (OR = 1.34; 95% CI 1.24–1.44) (29). Chronic stress exposure can trigger the activation of the hypothalamic-pituitary-adrenocortical axis, and enhance the secretion of cortisol. The increase of cortisol promotes urinary calcium excretion via competing with aldosterone at the renal intracellular level, finally leading to stone formation (30). Fourth, individuals with food insecurity are more likely to consume a variety of energy-dense foods (31). A previous study published by Nackers et al. found that low/very low food-secure households demonstrated greater access to microwaveable or quick-cook frozen foods (32). Similarly, Sharkey et al. reported that food insecurity was associated with an increased intake of total calories from added sugars (33). An energy imbalance between consumed and expended calories contributed to the development of obesity, which was considered an important risk factor for stone formation (26).

Our study has several strengths. Foremost, we conducted a correlation study by utilizing a large nationally representative sample of American adults. It is one of the largest cohorts and is well-defined. Furthermore, the food security status of participants was assessed by the standardized Household Food Security Module, which has been validated previously (9, 12, 34). Additionally, two multivariate logistic regression models adjusting for potential covariates including demographics, socioeconomic factors, physical activity, diabetes, serum uric acid, and intake of sugar, water, and vitamin B6 were conducted to obtain reliable results. However, the current study still has some limitations. Most notably, we cannot infer causality because of the nature of the cross-sectional survey study. In addition, the assessment of kidney stones was based on a self-reported questionnaire, and the results were vulnerable to recall bias. However, the validity and accuracy of this self-reported questionnaire have been confirmed in the previous study (20, 21, 35). Then, NHANES did not record the data regarding the time and type of kidney stones. Besides, the assessment of food insecurity status was made at the household level, but kidney stones were evaluated at the individual level. Nevertheless, a previous study published by Banerjee et al. demonstrated that household food insecurity had effects on almost all individuals in the household (34).

## Conclusion

Our study revealed that food insecurity may be positively associated with a higher likelihood of kidney stones and kidney stones recurrence. Our findings highlight applying public policies to improve the accessibility and quality of food for the prevention and management of kidney stones.

## Data availability statement

The original contributions presented in the study are included in the article/Supplementary material, further inquiries can be directed to the corresponding author.

## Ethics statement

Written informed consent was provided for each participant and the National Center for Health Statistics Research Ethics Review Board approved the project. The patients/participants provided their written informed consent to participate in this study.

## Author contributions

WW and XL proposed the conception and design. XL and YS provided administrative support. WW, XL, and YS supplied the study materials. XL and XW collected and assessed the data. XW analyzed the data. All authors contributed to the article and approved the submitted version.

## Funding

This study was supported by the new clinical technology in West China Hospital of Sichuan University (Grant No. 20HXJS002); Special Fund for Science and Technology Cooperation between Sichuan University and Panzhihua (2018CDPZH-29).

## Conflict of interest

The authors declare that the research was conducted in the absence of any commercial or financial relationships that could be construed as a potential conflict of interest.

## Publisher's note

All claims expressed in this article are solely those of the authors and do not necessarily represent those of their affiliated organizations, or those of the publisher, the editors and the reviewers. Any product that may be evaluated in this article, or claim that may be made by its manufacturer, is not guaranteed or endorsed by the publisher.

## Abbreviations

NHANES: National Health and Nutrition Examination Survey
OR: odds ratio
CI: confidence interval
U.S.: United States
COVID-19: coronavirus disease 2019
NCHS: National Center for Health Statistics
PIR: ratio of family income to poverty
BMI: body mass index.
