# Supplementary material for: Association between food insecurity and kidney stones in the United States: Analysis of the National Health and Nutrition Examination Survey 2007–2014
Source: Front Public Health. 2022 Nov 9;10:1015425. doi: 10.3389/fpubh.2022.1015425 (PMC9682121; doi:10.3389/fpubh.2022.1015425)
Supplement: Supplementary file 1 [file Table_1.DOCX]

Table S1. Questions assessing the adult food security in the U.S. Food Security Survey Module.

| Questions about food conditions of the household as a whole |
| --- |
| 1. “We worried whether our food would run out before we got money to buy more.” Was that often, sometimes, or never true for you in the last 12 months? |
| 2. “The food that we bought just didn’t last and we didn’t have money to get more.” Was that often, sometimes, or never true for you in the last 12 months? |
| 3. “We couldn’t afford to eat balanced meals.” Was that often, sometimes, or never true for you in the last 12 months? |
| Questions about food conditions of adults in the household |
| 4. In the last 12 months, did you or other adults in the household ever cut the size of your meals or skip meals because there wasn’t enough money for food? (Yes/No) |
| 5. (If yes to question 4) How often did this happen—almost every month, some months but not every month, or in only 1 or 2 months? |
| 6. In the last 12 months, did you ever eat less than you felt you should because there wasn’t enough money for food? (Yes/No) |
| 7. In the last 12 months, were you ever hungry, but didn’t eat, because there wasn’t enough money for food? (Yes/No) |
| 8. In the last 12 months, did you lose weight because there wasn’t enough money for food? (Yes/No) |
| 9. In the last 12 months did you or other adults in your household ever not eat for a whole day because there wasn’t enough money for food? (Yes/No) |
| 10. (If yes to question 9) How often did this happen—almost every month, some months but not every month, or in only 1 or 2 months? |
